# The Problem of Substitutes

**Published:** 1917-05-05

**Authors:** 


### FOOD IN WARTIME.*

### The Problem of Substitutes.

The Local Government Board has done good service in laying down rules for the guidance of Guardians in substituting other articles for bread, meat, and sugar in the dietary of workhouse inmates, and the conclusions they have arrived at may with advantage be studied and copied by all who have to cater for large numbers.

The recommendations are as follows :—

1. In altering a diet three-quarters of the former bread allowance must still be given as bread, and half the former meat allowance must continue to be given as meat. This is a very important principle for large households. In private houses it is fully permissible to try experiments, to give up meat altogether for a time, and contrive by well-thought-out schemes of alternative dishes to reduce the bread ration to half. All honour to those who treat their ration as though it were excessive, and have the wit to make use of the numerous other foods available. But experiments of this drastic nature are entirely out of place in victualling persons who are in any sense subordinates, and must take what is given them. It is a safe rule in such households to provide at least three-quarters of the quantity of bread and at least half the quantity of meat to which the persons catered for were accustomed before the rations came into force.

We venture in this connection, however, to remark that the average amount of meat and bread consumed per head in an institution is (or was) very often considerably higher than the amount actually consumed by any individual. Waste and muddle are large eaters, and it need not follow, because the meat ration is reduced from over 5 lb. per week per head to 2½ lb., that anyone need feel the pinch. For the new energy and resource which housekeepers are displaying in this hour of need does in many cases make every article go half as far again as in former days.

2. According to the new scale of substitutes, for every pound of bread or every ¾ lb. of flour over and above three-quarters of the usual bread allowance may be substituted ¾ lb. of barley, oatmeal, rice, sago, tapioca, or maize-meal, such as cornflour, hominy, etc., or 5 oz. of butter, margarine, or other fat.

### The Benefits of Variety.

If this rule were observed universally, whether in peace or war, nothing but benefit could accrue to the consumer. Six pounds of bread per head per week was a very common average rate of consumption in workhouses before the new rations came into force, and this average indicated that intense monotony of diet prevailed. In such households, according to the new scale, the bread and flour average will now be reduced to 4 lb. per head, and 1½ lb. of other cereals to make up will be substituted for bread. The intelligent use of the cereals enumerated above would introduce a much needed element of variety and liberate the inmates from the monotony of perpetual bread-eating. We are convinced that the extra variety is imperatively called for in the interests especially of children, and that the Birmingham Guardians are quite mistaken in believing children must have 6 lb. of bread per head per week. Too much bread makes children puffy and lethargic, and a change is productive of increased vitality.

Most cereals require the addition of a little fat to make them appetising. In making a sago or tapioca pudding a little margarine is a great improvement. Savoury rice or sago requires a good deal. And generally it may be taken for granted that substitutes for wheat flour, except in the form of porridge, are improved by a "nut" of fat, used in cooking. Hence the suggestion of the Local Government Board that 5 oz. of butter, margarine, or fat may be substituted for ¾ lb. of bread opens up great possibilities for the cook. For if the quantities are pooled, every household can substitute a proportion of fatty substances in addition to a proportion of cereals, and thus the cook will have at her disposal plenty of good material, and the dietary will gain all round.

### Utilising the Nitrogenous Vegetable Foods.

3. For every pound of meat, uncooked, without bone, provided half the quantity of meat usually allowed per week remain as before, the dietary provides for a substitution, if desired, of 5 oz. of cheese, or 3 oz. in each case of dry beans, lentils, or peas.

Here, again, it is imperative in the interests of the diners that the substitutes shall be pooled and used in combination. Suppose the peas, beans, or lentils were served plain-boiled one day and dry cheese the next, an exceedingly unsatisfactory meal would be offered on both occasions. But if among every 100 diners 75 per cent. of pulse and 25 per cent. of fat were used for the preparation of dinner, everyone would enjoy the repast. Served in the form of a pie with potato or rice covering, and flavoured with cheese, or in one of a dozen other ways in which cheese is a necessary ingredient, peas and beans make a satisfying and most nourishing dish. It is to be feared that owing to want of instruction the pulse varieties of food are scorned by the cook, and in consequence tend to be endured rather than enjoyed by diners. It is only necessary to visit any popular restaurant at the present day to realise the vast possibilities from the culinary point of view which reside in the alternative articles, used collectively, which make up a modern workhouse diet-table. It is certainly very disheartening to observe how extravagantly, yet how monotonously, the inmates and staff alike are catered for in Poor-Law institutions. Reduce their rations to raw material, and they are seen to consist of the same ingredients as are employed in hotels and restaurants which cater for the rich. Nothing, in short, is lacking but intelligent supervision and instructed cooks.

### The Art of Breadmaking.

Some old-fashioned economists are wondering whether alternatives to bread may not result in an increase of cost. The remedy is at hand if this be so. The art of breadmaking, too long neglected in the South of England, would, if revived, more than make up for the extra cost of substitutes. With bread at 1s. a quartern, it would be a saving worth making to reduce its cost to 2d. a pound, as is done in certain institution bakeries. In many small houses the bread is now being baked at home, and with a larger admixture of barley flour than is imperative under the regulations. This plan might be carried out everywhere. But in too many directions energy still lacks, although the war lasts on.

* Previous articles appeared February 24, March 10, 24, and 31, and April 14 and 21.

